# The Physiology of the Domestic Animals

**Published:** 1889-12

**Authors:** 


*The Physiology of the Domestic Animals.* By ROBERT MEADE SMITH, A.M., M.D. Pp. 938. Philadelphia and London: F. A. DAVIS. 1889.

It is a curious and suggestive fact that, while several excellent French and German works on what may be called comparative physiology have been in existence for a number of years, this is the first one which has appeared in the English language; and for this we are indebted to America.

Written originally by Professor Meade Smith for the use of his students in the veterinary department of the University of Pennsylvania, the work, however, appeals to all who are interested in physiology. The author has performed his task exceedingly well. In only a very few of the numerous departments can the doctrines enunciated be taken exception to; while in several sections—notably those on digestion, respiration, circulation of blood and animal movement—a very high order of merit in scientific description is maintained. The work is illustrated most liberally and admirably, and is got up in excellent style.

